# Prioritizing precision: guidelines for the better use of population descriptors in human microbiome research

**DOI:** 10.1128/msystems.00640-25

**Published:** 2025-09-23

**Authors:** Nicole M. Farmer, Amber Benezra, Katherine A. Maki, Suzanne L. Ishaq, Ariangela J. Kozik

**Affiliations:** 1 The Microbes and Social Equity working group, Orono, Maine, USA; 2 Nova Institute for Health 652687 , Baltimore, Maryland, USA; 3 Science and Technology Studies, Stevens Institute of Technology 33694, Hoboken, New Jersey, USA; 4 Division of Pulmonary and Critical Care Medicine, Department of Internal Medicine, University of Michigan 173243, Ann Arbor, Michigan, USA; 5 Department of Molecular, Cellular, and Developmental Biology, University of Michigan 118715 , Ann Arbor, Michigan, USA; Johns Hopkins University Bloomberg School of Public Health, Baltimore, Maryland, USA

**Keywords:** responsible conduct of research, biopolitics, ethical research, racialization

## Abstract

Microbiome science is a celebration of the connections between humans, our environment, and microbial organisms. We are continually learning more about our microbial fingerprint, how each microbiome may respond to identical stimuli differently, and how the quality of the environmental conditions around us influences the microorganisms we encounter and acquire. However, in this process of self-discovery, we have utilized socially constructed ideas about ourselves as biological factors, potentially obscuring the true nature of our relationships to each other, microbes, and the planet. The concept of race, which has continuously changing definitions over hundreds of years, is frequently operationalized as a proxy for biological variation and suggested to have a real impact on the microbiome. Scientists across disciplines and through decades of research have misused race as a biological determinant, resulting in falsely scientific justifications for social and political discrimination. However, concepts of race and ethnicity are highly nuanced, inconsistent, and culturally specific. Without training, microbiome researchers risk continuing to misconstrue these concepts as fixed biological factors that have direct impacts on our microbiomes and/or health. In 2023, the National Academies of Sciences, Engineering, and Medicine released recommendations on the use of population descriptors such as race and ethnicity in genetic science. In this paper, we posit similar recommendations that can and must be translated into microbiome science to avoid re-biologizing race and that push us toward the goal of understanding the microbiome as an engine of adaptation to help us thrive in a dynamic world.

## INTRODUCTION

Microbiome science can be a model for the interconnectedness of all things (1). We use microbiome science to discover how we relate to our environment, what is modifiable or not within our biological selves, what it means to function well, and how we can implement solutions to a rapidly changing environment. The microbiome is an engine of adaptation for ourselves and our environments; however, we limit the potential of microbiome science when we use artificial categorizations or social hierarchies as a proxy for biology. Recognition of the relevance of hierarchical thinking to the practice of science across biomedical disciplines has spurred multidisciplinary efforts to address long-overlooked factors contributing to health injustice. One such effort is the recently released “Using population descriptors in genetics and genomics research” report from a working group in the National Academies of Science, Engineering, and Medicine (NASEM) (2). The NASEM report addresses a critical issue–the inconsistent use of population descriptors in genomics research that conflate concepts like race, ethnicity, and ancestry and often lead to flawed interpretations and assumptions that lend scientific support to biological determinism or essentialism. The NASEM report extensively describes how ideas of racial categorizations came to be, ultimately defining race as “fundamentally a socio-politically constructed system for classifying and ranking human beings according to subjective beliefs about shared ancestry and innate biological similarities” (2). We also adopt this definition of race, where race is not a valid biological category, and the use of social definitions of race to organize biological differences is not substantiated by scientific evidence (2–4). When we speak of “race” in this paper, it is always referring back to this idea that race is socially defined.

NASEM brought together experts in African and African American studies, family medicine and community health, epidemiology, biostatistics, biomedical informatics, anthropology, sociology, medical humanities and ethics, law and sociology, and civil rights to generate and review the content of the report. They set out best practices and guiding principles for genetics and genomics research, and the committee states that their report “also provides a foundation and common vocabulary for researchers and other relevant parties to engage in future decision making for contexts that may not be addressed directly in this report.” Although not generally considered central in the study of human genetics/genomics, microbiome research is informed by our understanding of human genomics while also integrating studies of environments, relationships, and other host-associated factors. Within microbiome science, the role for all or one of these population descriptors has been discussed or reviewed regarding how human host differences are measured and represented as a relevant factor to microbiome composition, diversity, and function (4–7). Here, we discuss the guidelines presented in the NASEM report and their implications for the study of human microbiomes. This is a first step for microbiome scientists to incorporate the NASEM guidelines into their work. We outline three urgent calls to action for microbiome science with the objective of generating a shared, equitable, and more rigorous approach. The history of racialization and typological thinking about differences between human groups in genetics research has produced unethical and invalid findings, which have hindered the advancement of science. As the field of microbiology moves forward to address issues of health, environment, and society, it is imperative that we as scientists begin to enact changes in our disciplines that reshape how human microbiome studies are “conceptualized, conducted, and interpreted.”

Race is not a fixed or measurable trait, although it is often used as an oversimplified categorical factor. Racialization, the systematic construction of racial groupings, was used to create a system of human hierarchy. At its core was a desire to differentiate a whole (the human species) into parts with ranked degrees of worth. In the 1730s, a Swedish naturalist, Carl Linnaeus, devised a system that showed how living things are related to one another (8). He divided the human species into four varieties: European, American, Asiatic, and African–despite never having traveled outside of Scandinavia or having met the populations he classified. His classification of different racial groups as different, distinct varieties of humans laid the groundwork for others to use science and classification methods to try to justify discrimination through perceived biological differences (8). Each of Linnaeus’s racial descriptions embodies a stereotype: for “color,” he used red, white, yellow, and black, and included very problematic descriptions of other characteristics to imply that skin color determined personality and intelligence (8). Because of Linnaeu’s reputation as a naturalist, his work became the source reference for others to continue racialization, and although Linnaeus’s categories are not what we use today, we still participate in creating divisions to classify groups of humans based on observable traits like skin color. In microbiome science, this has led to the assumption that these divisions somehow manifest as biological determinants of the microbiome (e.g., references 9, 10; contrarily in references 11–13; and discussed broadly in references 4, 14).

The boundaries of racial categories, along with the definition of race itself, continue to morph over time in response to changing social and political needs and attitudes (3). Thus, using it as a shorthand for meaningful genetic variation has always been problematic. This is not to say that race should be invisible to microbiome scientists or seldom discussed; on the contrary, we argue that more attention should be paid to the use of race in microbiome studies, but carefully. Race must be analyzed for what it can actually tell us, framed by a clear understanding of what it cannot. Here, we provide guidance, similar to that in the NASEM report, to help researchers understand the context of racial categories: when race provides useful framing and when it is irrelevant to a microbiome study, how to discuss it in manuscripts, how to replace it with measurements of more specific factors, and how to improve the rigor and impact of microbiome research through interdisciplinary collaboration.

## WE CAN DO BETTER: MICROBIOME RESEARCH SUFFERS CRITICAL METHODOLOGICAL GAPS FOR CONSIDERING THE ECOSOCIAL REALITIES THAT IMPACT MICROBIAL EXPOSURES

Studies identifying race-based, “racio-ethnic” or “ethnicity-influenced” differences in microbiome diversity (alpha, beta) and composition (species, strain, and taxa) have emerged, discussed elsewhere (5), with large observational data and mechanisms underlying these differences rarely identified (beyond suggesting the identified taxa are mediators). Furthermore, socio-environmental variables beyond race or ethnicity that could explain their findings are not reported, and potentially important but unmeasured factors are often only briefly mentioned (9). Until recently, microbiome research has been limited by technological constraints, precluding an in-depth investigation of all the potential drivers of microbiome-associated biological processes and accurately understanding potential pathways and mechanisms. As our ability to combine sequencing, culturing, microscopy, metabolomics, and other methodologies improves, the field demands that researchers provide or posit the mechanism behind the structure and behavior of microbial communities they are describing. Host biological descriptors (i.e., age) and behavioral variables (i.e., diet) are required information to describe the population and potential drivers of variance in microbiome studies, but we cannot assume that these variables are homogeneous or act universally on all participants.

As a holobiont, humans have co-evolved with their microbial passengers to share genetic origins and functions. From a holobiont-based lens of human-microbe existence, we recognize that human microbiota has a role in our physiologic functions and health and that this interconnection may be representative of genetic co-evolution, thus necessitating a potential line of inquiry between human genetics and the microbiome. Nevertheless, the rate of bacterial evolution (through within-genome mutations and horizontal gene transfer) is several orders of magnitude faster than humans, as bacterial and archaeal populations can acquire mutations or exchange genes within hours while the human germline changes over millennia (15, 16). Although polymorphisms in host immunity-related genes (e.g., human leukocyte antigens [17], pattern recognition receptors) can modulate which taxa thrive in microbial niches (18), large population-based cohorts demonstrate that such loci explain only a small fraction (<10%) of between-person microbiome variation, whereas contemporary ecological exposures (i.e., diet, medications, pollutants, built environment [11, 19]) account for the majority of host microbial adaptability (20, 21). Therefore, host genetics may influence different host-associated layers of selection but are unlikely to be a direct and consequential one-to-one (i.e., “linear”) driver of microbial evolution. As detailed in the NASEM report, although geographic-location-based genetic variants exist, which are associated with past or present local environmental adaptation, these alleles are often not widely distributed within the human genome except for incidental cases such as the Duffy null allele and susceptibility to malaria (22). The microbiome studies that imply there is a role for host genetic differences between races (or ethnicities) are making the assertion that the well-reported minimal genetic variation between races (ethnicities) fits within broad categories defined by race, and furthermore that these small variations then account for gross microbiome changes in composition and diversity. Research on the connections between human ecology and microbes within geographic locations of people who have variation in allele frequencies, but who fall within socially defined racial groups, such as sub-Saharan Africa (21), may provide demonstrable insight into microbe and host overlap and assist in refuting the use of racial categories, such as Black, when the inference is that there is a racial-group-aligned genetic host-microbe connection.

Based on the above, we offer a view that has been widely called for in medicine and human research (5, 23, 24): that differences identified between socially defined racial groups in microbiome community characteristics occur because of the well-documented and extensive variation in the environmental and social drivers that can modulate the human microbiome. Thus, a more deliberate and precise quantification of social/environmental variables will allow researchers to think “below the surface” to identify structural drivers that impact social and environmental modulators of the microbiome. Relevant examples can include quantifying the stress caused by racism on inflammation or blood pressure (25–27), as well as air quality or shade indexes in neglected neighborhoods and the effect on lung and gut microbiomes (28). These environmental and social factors have been documented to be heterogeneous within and similar across race/ethnic groups. Therefore, more deliberate and precise quantification of variables to operationalize the relevant social and environmental factors in microbiome research, versus relying on racial groups as a nonspecific proxy for these factors, will allow quantification of the actual degree of factor-specific microbiome modulation. When biological differences are hypothesized to contribute to microbiome differences, we suggest underlying mechanisms, consistent with the ecosocial theory, are more likely to be responsive biological pathways, such as epigenetics and inflammation. Social and environmental factors also influence reactivity of underlying responsive pathways (and their subsequent effects on the microbiome), but biological variation may drive the degree and duration of the pathway response.

In addition to human genetics as a potential mechanism for explaining differences in the microbiome between groups, the concept of “heritable” taxa has been proposed as a mechanism based on the identification of certain taxa, such as Christensenellaceae, between racial groups. Misconceptions regarding the validity and measurement of heritable taxa in people have been discussed elsewhere (29, 30), and data supporting the mode of heritability in people are not conclusive: only a few bacteria transfer by direct vertical transmission, there is a negative association between offspring age and the percentage of bacterial strain-sharing between the birthing parent and offspring, and the exponential increase in gut microbiome bacterial richness in the first years of life suggests microbiome community characteristics are driven by environmental exposures rather than genetic determinism (31–33). Moreover, conflicting data regarding lifestyle or social contact are reported that can impact vertical or horizontal transfer (e.g., breastfeeding or cohabitation [32]). Importantly, the link with racial group differences for heritable taxa suggests that there are singular or homogeneous lines of inheritance within current racial groups. For example, within the United States Census Office of Management and Budget, only individuals categorized as “Black” or “Asian” racial groups are held to geographic ancestry-based categories, whereas individuals categorized as “White” may be from Europe, or the Middle East Asia, or North Africa. Furthermore, the suggested link to heritable taxa negates the role of social groups and social networks within racial groups, along with the influence of household and community cohabitation structure on strain-level bacterial transmission. Taken together, exposures throughout the environment in early life through adulthood facilitate persistent bacterial colonization, and race-based classification is not sufficient to quantify the extent or nature of these influential exposures. Linking socially defined race with heritable traits suggests that there are homogeneous racial lines of inheritance, but race is not singularly defined.

## HOW DID WE GET HERE: THE CONCEPT AND DERIVATION OF RACE IN SCIENCE

Science has participated in building global racialized systems of oppression, but especially scientists in Europe and North America (8). Familiar racial classifications like the Anglo-European system did not exist prior to explicit requests from governing institutions to create them in the 1600s–1700s (34, 35). Discrimination and stereotypes existed prior to categories, but they were based on factors such as country of origin, religion, or culture, not so-called “scientific” distinctions. Defining people as “out-group members” different from one’s own group is theorized to have begun as a protective behavior from potential pathogenic exposures (36, 37). Under this protective response, differences between groups were based on an out-group possessing a lack of customs, practices, and behavioral norms (36–38). Noticeable physical differences between groups were recognized but not ascribed to be representative of innate, biological, or hierarchical differences. In contrast, approximately 500 years ago, when Europeans embarked on an expansion of land and categorizing phenomena found in nature, “race” developed as an idea to characterize different groups as fundamentally different types of humans, thus introducing typological categories of humans (22, 39). With the advent of these categories came the notion that differences were reflective of nature and thus were innate and immutable. Ultimately, acceptance of these categories occurred within the European Christian religion, laws restricting rights for people of particular non-European races were created, and the utilization of science to justify the natural order of races was initiated. If humans could be viewed as biologically, immutably different, one could argue that not all levels of the hierarchy deserved the same rights.

In the 18th century, the scientific discovery of genetic units of inheritance led to further connotations presented on the connection between race and divine order through the race-based contentions of monogenism (divine creationism of one genetic line from the Bible) and polygenism (the theory that each race had its own genetic origin) (20). The indoctrination of race within science, however, started a century before notions of inheritance in the 18th century. British colonial institutions sought validations of colonial efforts through curating scientific projects to investigate the origins of race. The role of these institutions in dictating scientific efforts and direction on the concept of race can be inferred from the formation of the Royal Society (England), the Council of Foreign Plantations, and the slave-trading Royal Adventurers in Africa all within one year (1660), in which several shared members existed across the institutions (33, 40). Shortly after its inception, the Royal Society initiated projects to evaluate skin color, the primary trait of the time attributed to racial differences. These projects focused on the skin of Africans, “Mongoloids,” and the Moors. Several projects argued that the origin of “black” or darker skin was inherently different than white skin and further imputed inferences from “the great chain of beings,” which stated that African people were naturally not divine brethren of Western Europeans (33). One project funded by the Royal Society proposed that Africans possessed a different type of bile that permeated outward to the skin or contained pigmented sweat glands that were responsible for skin color. Ultimately, the scientific projects from the Royal Society contributed to an evidentiary justification for the social, colonial, and political derivations of race, laying the foundation for the institution of racist behaviors, policies, laws, and norms in the Americas, Asia, and Africa.

As stated by Koslofsky, “The approach to blackness promoted by the Royal Society was to affix dark skin firmly in African blackness and bodies and to tie it to deeper or more innate characteristics” (41). These emerging scientific dogmas on race and skin color as a representative marker were challenged through the utilization of the microscope. The microscopist and cellular physiologist Antoni van Leeuwenhoek, himself a member of the Royal Society, was requested by the Royal Society to investigate skin color (33, 41). Through his one-eyed microscope, he made a different determination than most scientists at the time: he reported that skin color was determined only by differences in “igment scales” rather than an intrinsic chemical difference (41). He also reported that “whiteness is as much a matter of growth and development as blackness.” However, his work—which did not identify a more than “skin deep” meaning of race—was not widely publicized by the Royal Society (33). As we now know, in 1683, Antoni van Leeuwenhoek would provide the first known description of bacteria using his microscope, thus sparking the field of human microbiome research.

In his 1795 book *On the Natural Variety of Mankind*, Johann Blumenbach, a German anatomist and naturalist, coined the term Caucasian (8). Blumenbach divided all humans into five groups, defined both by geography and appearance. In his order, they are: Caucasian, for the light-skinned people of Europe and nearby parts of Asia and Africa; Mongolian, for most other people from Asia, including China and Japan; Ethiopian, for the dark-skinned people of Africa; American, for most Native populations of North and South America; and Malay, for Polynesians and Melanesians of the Pacific and Aboriginal Australians. Blumenbach changed the original racial classification developed by Linnaeus by adding the Malay category. He also changed the principle of human order from being geographical to a hierarchy of worth. Although he did not believe that different races were created separately and did not agree with enslavement by reason of race, Blumenbach based his hierarchy on how beautiful he thought each group of people was, thus instituting ideals of a Caucasian standard. In America, at around the same time, Samuel Morton was a physician practicing in Philadelphia and was considered a great scientist. He supported the idea that races were different species through his work measuring human skull volumes (42). Morton believed that a larger skull volume meant more intelligence. He then used this to rank human intelligence. His measurements found that modern Caucasian skulls (the term he used for those of European descent) had the highest volume. Next were skulls from China, followed by Africa, South Asian, and American Indian skulls. Morton’s work was used to directly support the system of American chattel slavery.

These scientists influenced not only how humans were categorized scientifically but also within society and ultimately influenced the codification of some race-based laws. If Africans were not of the same species as Caucasians, then enslaving them was more easily explained and excused. Samuel Cartwright, a New Orleans physician and supporter of slavery (8), in 1851 published his “Report on the Disease and the Physical Peculiarities of the Negro Race” (https://www.pbs.org/wgbh/aia/part4/4h3106t.html), in which he argued that high rates of physical and mental illnesses afflicting black persons were products of the supposed biologically inferior mental capacity of the “black race.” In this report, Cartwright introduced what he called “drapetomania,” known as the “disease causing slaves to run away.” Cartwright viewed drapetomania as a mental “illness” that could be beaten or tortured out of those who resisted enslavement. Cartwright’s work influenced the reasoning of Justice Roger B. Taney in the Dred Scott decision of 1857, which determined that Black individuals did not have the right to American citizenship (8). Dr. Cartwright’s other contributions to science include race-based differences in lung capacity, which still impact race-based lung function calculations in the present day (43).

The outcome of these and many other racist ideas was continued in society with the eugenics movement—a theory that the human population could be improved or human evolution accelerated by selective breeding. The U.S. eugenics movement spread its message through incredibly effective science communication at state fairs, educational institutions, and professional conferences across the country (44). The movement was thriving and funded and championed by many well-known scientists from the 1880s to the late 1930s, and it influenced domestic and immigration policy (44, 45). There were meetings of scientists and thinkers, where eugenics was considered a progressive, mainstream, scientifically sound ideology and was used to inform genocidal policies and practices.

As a result of the contributions from these and other scientists, the framework by which we define and examine human beings was shifted from evaluating observable traits in the context of ancestry, environmental conditions, or social exposures to discrete categories in which similarities across categories are diminished, as are differences within categories. A further result is the premise that human traits and biology occur inherent to the categorical descriptor, and not as a result of socio-ecological environmental exposures and contexts, such as living conditions and responses to social interactions. Human genetic and microbiome variation is, instead, dependent on the complex intersection of social, biological, and environmental factors. Different fields of science, as well as anthropology, sociology, bioethics, and public health, disagree about what race even is; however, race is continuously used to describe groups of scientific subjects. The NASEM “population descriptors” paper, as well as authors of this paper, has discussed at length the problems with using “race” as a proxy for other variables—variables like what neighborhood someone lives in, whether they can afford to go to the doctor, and if they live with daily racial discrimination—that play a real and enduring part in biological and health outcomes (6, 7, 46, 47).

**Fig 1:**
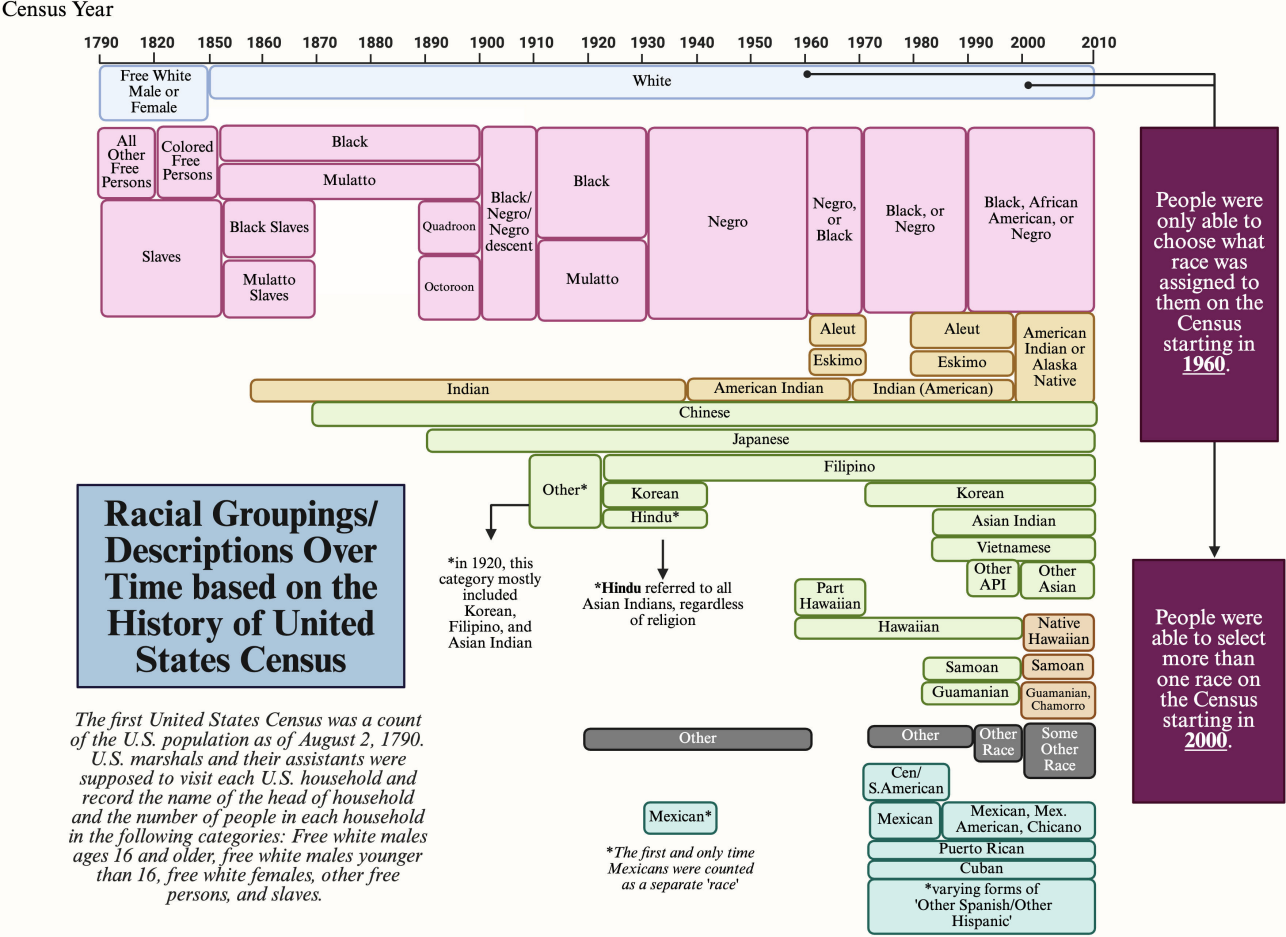
Racial and ethnic Census labels from 1790 to 2010 (based on images from reference 49). Created in BioRender (A. Kozik, 2025, https://BioRender.com/8ds0s7p).

Race may be self-identified, socially assigned, or socio-political; it is nationally and regionally relative; and can be combined with or exclude ethnicity (i.e., Hispanic/LatinX) (48). The boundaries of who is considered “Black” in the United States is different from “Black” in Belgium, South Africa, or Brazil. Through 1950, census-takers commonly determined the race of the people they counted. From 1960 onward, Americans could choose their own race. Starting in 2000, Americans could include themselves in more than one racial category. Before that, many multiracial people were counted in only one racial category. The timeline reveals some interesting shifts. For instance, the category “other race” vanished between 1850 and 1900 and then returned as an option in 1910. Asians were acknowledged in 1860, when “Chinese” first appeared as a category. By the 1900s, the sub-categories within “Asian” became more detailed—although not all were quite accurate. From 1920 to 1940, for example, “Hindu” appeared under this umbrella as a separate race, presumably meant to be checked by all South Asians at the time—Hindus, of course, are the dominant religious group in India and Nepal (https://www.bbc.co.uk/religion/religions/hinduism/), not a racial group (Fig. 1).

Furthermore, disparities in health and disease typically attributed to race have been shown to be the result of systemic bias, oppression, racism, and discrimination, not “just biology.” “Racism shapes social experiences and has biological consequences, and that race is not a meaningful scientific construct in the absence of context” (50). For example, poor maternal outcomes for Black Americans are not because of a deterministic difference in Black maternal biology but instead show how structural racism results in poor care, misdiagnoses, and generational trauma (51, 52). Similarly, Black mothers’ vaginal microbiota is not racially, but is rather socially, determined (53, 54).

Social science research has shown the danger of presupposing the biological essentialism of race (3). The social and ethical consequences can be medical racism, implicit bias, and a continued legacy of health disparities. The scientific consequences can be inaccurate data, variables based on imprecise and false categorizations, and results that reinforce racial stereotypes and support continuing inequities. These social determinants of health have real impacts on biological outcomes, and the health disparities they cause are sometimes erroneously attributed to race (50, 55).

Translational results of human microbiome science models themselves on personalized medicine—characterizing states of human health and illness microbially, dependent on individual biosocial factors like nutrition, diet, and environment. In this sense, microbes should be raceless. However, race, gender, and other socialized categories have become central to choosing and classifying subjects for microbiome research (5)—and racial difference, rather than environmental and structural drivers, erroneously becomes the explanation for microbial difference, which is not ameliorated when authors use racial categories in the title and abstract and later explain that these are actually proxy terms for social factors (9, 56). Furthermore, these broad categories lack intersectionality (57), and the reuse of data from databases often means the meta-analysis fails to mention if the samples were obtained from the sample geographic area (57).

### Ecosocial theory as a path forward

Our biological systems are designed for adaptation to exposures, which can be social, environmental, and psychological. Thus, exposures are a key metric to evaluating biological systems at the individual and population level. However, the narratives of race that have carried into current-day science are oftentimes treated as an exception to our basic understanding of biological responses. The exception, and continued narrative, is that by “virtue of being Black,” by “virtue of being White,” or by “virtue of being ‘enter social group,’” we can expect to see a biological outcome. The challenge to this line of reasoning has been present for centuries and is explicitly stated in the works of the sociologist, Dr. W. E. B. DeBois. More recently, social epidemiologist Nancy Kreiger has articulated a new way of theorizing health, the ecosocial theory of disease distribution (ETDD), to understand, explain, and change population health. The first premise of ecosocial theory will sound familiar to those accustomed to thinking about microbial ecologies: every living being is necessarily and simultaneously an individual and part of a population shaped by its history and engages dynamically with its own and other species (58). As microbiome scientists, we know that our resident microbial communities take shape early in life and stay with us over the course of the lifespan. We have parallel concerns: energy, defense, and the creation of stable communities. As humans and hosts, we are at once in the environment and functioning as an environment. These authors also argue that the second and third premises of ETDD must inform the future of the field. (ii) Our bodies tell stories of our experiences*. “*Organisms live their phenotype, and this phenotype is not fixed but emerges through engagement with...social and biophysical features of the dynamic changing world we inhabit and alter.” (iii) The reason to analyze health inequities is “to illuminate how both injustice and equity can respectively shape people’s health and the health of the planet for bad and for good.” As a population descriptor, race tells us incredibly little about a person’s biology but considerably more about their experiences as a consequence of a system that used a racial system to control access to resources and shape environments, and as such, should instruct us to think critically about the ways we operationalize it in our research. Table 1 adapts ETDD to microbiome science, aligning each construct with concrete exposure pathways and analytic considerations that may help researchers move beyond attributing differences in microbiome communities to race and consider different factors that may drive heterogeneous microbial ecosystems.

**TABLE 1:** Translating Krieger’s ecosocial theory to human microbiome research: constructs, definitions, and implications for measurement^*a*^

Construct	Key components	Definition	Implications for microbiome research and example(s)
Embodiment	Societal conditions Dynamic ecological state Group relations Cultural practices and beliefs	The body (host + microbiome) continuously engages with social and biophysical contexts	Exposure metrics—not racial categories—should be the starting point. Example: neighborhood disinvestment can lead to limited access to fresh foods and alteration or reduction in nutrients reaching the gut
Pathways of embodiment	Social and economic (de)privations Affordances/discrimination Healthcare access Ecosystem health	Multiple, interacting routes through which positive and negative exposures are processed and integrated “inside the body”	Rather than attributing differences to “race,” map the socially structured exposures that may influence these pathways (e.g., food deserts, pollutant burdens, stress load) and test their microbial consequences
Cumulative interplay of exposure, susceptibility and resistance	Embodied exposures Conditioned responses Gene × environment interactions	Patterns of exposure accumulate over historical time and biological development	Examine how adverse childhood experiences and/or layered exposures across the life course may impact stress physiology. Example: chronic psychosocial stress from discrimination can lead to altered HPA-axis reactivity and increased intestinal permeability
Accountability and agency	Levels of power Individual/collective capacity to act	Capacity to change inequitable conditions and to generate explanatory research	Design studies and interventions that give agency to communities. Example: community-led urban gardening projects that improve produce access and diversify dietary fiber sources, impacting nutrient availability and the environment of the gut microbiome.
Lifecourse	Historical context Generational effects	Health and microbial trends are patterned by historically specific events and policies	Situate microbiome data within historical timelines (e.g., cohort birth year, policy eras)
Processes	Political-economic organization of resources and labor	Societal arrangements that allocate material conditions shaping exposures at every level	Question whether the microbiome is being shaped by market forces rather than intrinsic “racial” differences. Incorporate data on food systems, housing, and healthcare access when interpreting microbial variation.

^*a*^ Six core constructs of Nancy Krieger’s ecosocial theory of disease distribution in the context of microbiome, science adapted from Krieger (59) and Tomasso and Chen (60). For each construct, key components originally articulated in ecosocial theory, a microbiome-focused definition, and specific implications for study design, data interpretation, and the ethical handling of race and other population descriptors are presented. Abbreviation. HPA: hypothalamic-pituitary-adrenal (axis).

ETDD offers a new model for understanding these relationships by articulating a need to consider impacts at multiple societal levels, identify, and study pathways of embodiment and how existing and historical power dynamics shape them. Because the system of racial hierarchy in the United States was used to control resources and shape environments over centuries, we must recognize that the impacts traversed multiple levels of society, from the global to the individual, and that these impacts intersect to impact a person’s health. The mechanisms by which the forces at each level influence health are referred to as pathways of embodiment. Stated another way, these are the ways that social and environmental exposures have a direct biological impact. In microbiome studies, race is often discussed or presumed to function at this level, as an independent factor, a pathway that can elicit a biological response or predetermine a biological state. However, race is actually a power dynamic, something that modifies the exposures themselves. As such, rather than comparing the microbiomes of racial group A to racial group B and concluding a “race-associated difference,” we should instead compare the social and environmental conditions of group A and B and discuss whether those conditions could be shaped by exploitative and/or oppressive inequalities (i.e., racism). ETDD emphasizes the importance of considering spatial and temporal contexts in our analyses. The recognition of temporal and historical drivers is critical, especially in the study of U.S. populations, because some individuals who are alive right now (at time of writing, anyone older than 60) were alive during eras where actual policies and laws were enforced that structured social and environmental exposures in a way that intentionally disadvantaged people racialized as non-White.

## CALLS TO ACTION

**Fig 2:**
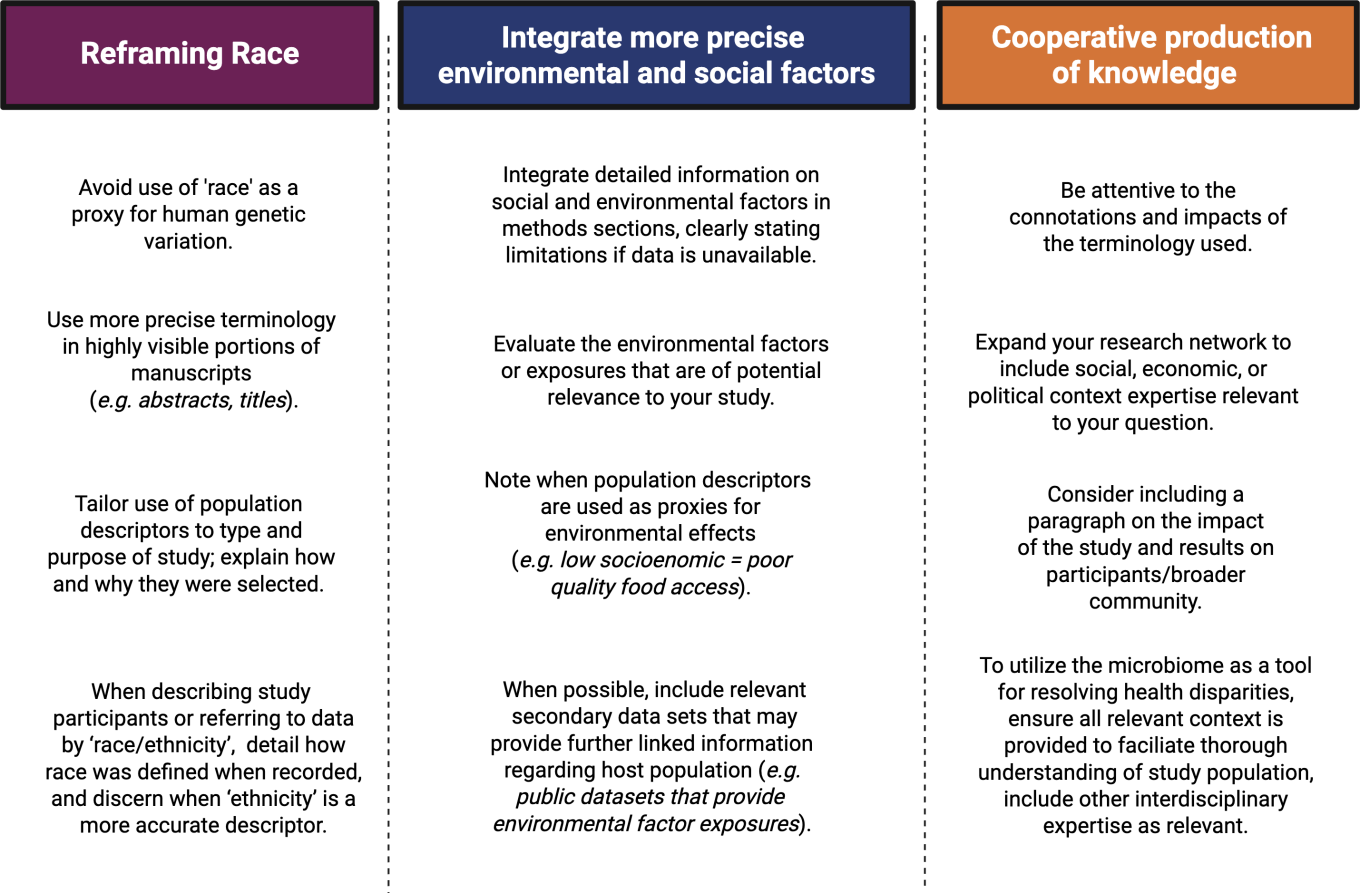
Summary of our three calls to action for microbiome science. These are modeled after the NASEM report’s three requisites: avoid typological thinking, measure environmental factors, and engage communities and participants. Created in BioRender (A. Kozik, 2025, https://BioRender.com/xp8k4ig).

Scientific discoveries and subsequent biomedical and technological advances have profound impacts on society, policy, law, economics, and medicine–influencing the types of questions being asked; determining which people are trained, equipped, and funded to do the asking; and what resources are available and to whom (61–63). Simultaneously, societal needs, pressures, and hierarchies shape the trajectory of scientific investigation. Science and society are inextricably linked in symbiosis, with the potential for both mutual benefit and harm. Science, and public understanding of science, is central to society and impacts policy, culture, the economy, and the law. In short, what we do as scientists, how we ask questions, how we communicate findings, and the way in which our science is interpreted have ripple effects through society. Our three Calls to Action for microbiome science are modeled after the NASEM report’s three requisites: avoid typological thinking, measure environmental factors, and engage communities and participants (Fig. 2).

### Reframing race

#### Do not use, invoke, or categorize according to race without a discussion of racism

*NASEM recommendation 1: researchers should not use race as a proxy for human genetic variation. In particular, researchers should not assign genetic ancestry group labels to individuals or sets of individuals based on their race, whether self-identified or not.*

*NASEM recommendation 6*: *researchers should tailor their use of population descriptors to the type and purpose of the study, in alignment with the guiding principles, and explain how and why they used those descriptors. Where appropriate for the study objectives, researchers should consider using multiple descriptors for each study participant to improve clarity.*

*“*It is time for us to reshape how genetics studies are conceptualized, conducted, and interpreted.” – Aravinda Chakravarti and Charmaine Royal, Cochairs, Committee on the Use of Race, Ethnicity, and Ancestry as Population Descriptors in Genomics Research (2)

The use of race as a proxy for human genetic variation is also a problem in microbiome research and has been reviewed extensively (3, 4, 6, 7, 64), and chapter 2 of the NASEM report extensively describes descent-associated variables as well as how they may be accurately applied (2).

When describing study participants or referring to data by “race/ethnicity,” researchers should detail how race was defined when recorded, discern whether they mean “ethnicity,” and explain the geographic sociopolitical context needed to understand/define this population. For example, individuals who self-identify as Hispanic/Latinx or are from the Caribbean may not uniformly relate to U.S. conceptualizations of the racial construct (65). In 1976, ethnicity was instituted by the U.S. Census as a self-identification category under the binary categories of Hispanic or non-Hispanic, ignoring that within racial categories, there may be ethnicities that are culturally recognized. This binary ethnicity method of categorization, which views race and ethnicity as mutually exclusive, introduces variation between respondents and discordance across studies. Alternatively, when not mutually exclusive, the selection of White or Black or Other for individuals of Latin descent prevents accurate data counting of these individuals in studies and precludes usage of heritage lines (i.e., Mexican, Cuban, and Puerto Rican), which is often a culturally preferred self-identity and may be relevant to ecosocial factors that impact the microbiome, such as diet (66).

The NASEM report highlights the appropriate use of race when conducting health disparities research. Identifying health disparities is predicated on the measure of racial and ethnic differences, as race is often understood to be the leading factor in health disparities. It is important to highlight that the sheer number of microbiota and multifarious conditions affecting microbiomes has made it difficult for research to establish a causal role for most nonpathogenic microorganisms in contributing to health or illness (67). Thus, although we can identify endpoint microbiome differences in states of health vs. disease, we cannot point to the starting microbiome differences as inherently good or bad. Rather, the microbiome may be changed as a result of the disease process or the social or environmental exposure. Therefore, presenting race-based differences in the microbiome only documents microbiome disparities but not necessarily health disparities. To utilize the microbiome as a tool for resolving health disparities, continued inclusion of self-identified individuals of diverse populations within microbiome studies is important, in addition to the measurement of disparate social and environmental exposures.

##### How to handle latent variables

The NASEM report contains a thorough discussion of how to identify social determinants of health and to more specifically define social and environment factors, as well as how to determine if there is an appropriate use of race and ethnic characterizations in specific microbiome research exploring the role of human microbiome composition, impacts of racism/discrimination, and racial/ethnic health disparities—similar to the NASEM report’s Table S1 (page 14) (2). Recognition of health disparities and the mechanistic factors, such as exposure to discrimination, socioeconomic status, and residing in socially vulnerable geographic regions, are germane to microbiome research as microbes may become markers of—and through metabolic or inflammatory processes related to ecosocial factors—contributors to these disparities (68, 69). Similar to Table 5-1 in the NASEM report (2), inclusion of the impacts of racism/discrimination and contextualization of racial/ethnic health disparities present within a selected study population may be applicable and should be measured, when possible, within microbiome studies. Examples for researchers based on the information provided in the NASEM report are presented below.

- Once the appropriate population descriptor is identified for the context of their study, apply group labels consistent with that concept to all study participants—for example, individuals of low or high socioeconomic status as a population descriptor.

- More than one descriptor may be appropriate, and studies may benefit from using multiple descriptors—for example, individuals of a certain age group who are of low or high socioeconomic status.

- Population descriptors are sometimes used as proxies for environmental effects; it should be explicitly noted when population descriptors are used as proxies in this way, and the rationale should be provided—for example, individuals of lower socioeconomic status who reside in certain geographic locations may be a proxy for areas with poor quality food environments that influence diet intake, which can modulate the gut microbiome.

To utilize the microbiome as a tool for resolving health disparities, continued inclusion of self-identified individuals of diverse populations within microbiome studies is important, in addition to the measurement of disparate social and environmental exposures. Microbiome research is slowly changing the field to increase inclusion, but this is a lesson for microbiome researchers (70). In 2021, the microbiome research community recommended using the STORMS checklist to standardize and increase the detail on describing methods and participants (71). The checklist is regularly updated (72), and as of August 2023, it makes no specific mention of race. We recommend that this and other human subjects resources integrate information for authors to determine if and how they should collect that data, and how to report it. Suggestions for microbiome researchers to consider population descriptor reporting and social and environmental factors in reporting their research are provided below in the context of the STORMS categories.

- Introduction
  - Within the background, provide rationale for using the selected host population, and for use of the population descriptors selected in relation to the research question(s)
- Methods
  - Provide data sources and measurements, including information about social and environmental factors, if available. If not available, clearly state the limitation to the methods from the chosen data set(s), especially if social and environmental factors are not included that may influence microbiome exposures or outcomes.
  - When possible, include relevant secondary data sets that may provide further linked information regarding the host population. This may include the use of public data sets that provide environmental factor exposures based on limited host residential and occupational information.
- Results
  - Present descriptive host data relevant to selected population descriptors
  - Report statistical analyses that include relevant host population descriptors, including social and environmental factors
- Discussion
  - Present the interpretation and generalizability in the context of the measured population descriptors
  - Present limitations of measured population descriptors, including when social and environmental factors were not or could not be measured.

### Do not create unsubstantiated hierarchies for “good and bad microbiomes”

*NASEM recommendation 2*: *when grouping people in studies of human genetic variation, researchers should avoid typological thinking, including the assumption and implication of hierarchy, homogeneity, distinct categories, or stability over time of the groups.*

*NASEM recommendation 3: researchers, as well as those who draw on their findings, should be attentive to the connotations and impacts of the terminology they use to label groups.*

A tenet of microbiome research is the classification of microorganisms and further categorization of microbiota to aid in understanding potential function (i.e., gram-negative, butyrate producers). These steps in classification and categorization aid the field in providing functional context, improving precision, and enabling meaningful use of data; identifying similarities and relationships; and proposing a mechanistic understanding. For example, it is important to highlight that the sheer number of microbiota, their situationally specific activities toward hosts, and multifarious conditions affecting microbiomes have made it difficult for research to establish a causal role for most microorganisms in contributing to health, illness, or neither (67, 73, 74). Host-microbe interactions are largely dependent on context, and microbes may have vastly different functions and byproducts depending on the ecological community they are exposed to, along with adjacent cooperative or antagonistic microbial processes. These broad generalizations of the whole microbiome community based on the identification of certain microorganisms as ubiquitously pathogenic lack specificity and are not clinically meaningful. Similarly, as microbiome researchers, we need to question the use of “race” as an explanatory variable or challenge ourselves and our peers to explain the mechanism by which race might be an explanatory variable, for example, by exploring the confounding roles of racism and stress (26, 75). When we think of people as categories rather than individuals, we tend to stereotype and think of everyone else as exceptions or outliers. But nurture and localized circumstances override this all the time (76, 77). Microbiome research is slowly changing the field to increase inclusion, but this is a lesson for microbiome researchers (70, 78).

Microbiome researchers are trained to interpret data in hierarchies or to identify preferred microbial communities; however, our studies often lack enough context or detail to do so with any accuracy. For example, more microbial diversity does not equate to better health in all situations; however, microbiome science typically assumes more richness is preferred in any situation (79). Similarly, microbiome studies may more frequently allude to the effect of lifestyle and behaviors on the microbiome as an example of host-microbe interactions, but studies almost never capture the structural factors behind behaviors, despite data showing that choice, or lack thereof (e.g. environmental constraints), insecurity of food, healthcare, housing, and personal products can affect the microbiome [27,28].

### Integrate more precise environmental and social factors instead of socially defined “race”

#### Do not use “race” as a proxy for mechanistic variables

*NASEM recommendation 4*: *researchers conducting human genetics studies should directly evaluate the environmental factors or exposures that are of potential relevance to their studies, rather than rely on population descriptors as proxies. If it is not possible to make these direct measurements and it is necessary to use population descriptors as proxies, researchers should explicitly identify how the descriptors are employed and explain why they are used and are relevant. Genetics and genomics researchers should collaborate with experts in the social sciences, epidemiology, environmental sciences, or other relevant disciplines to aid in these studies, whenever possible.*

**Fig 3:**
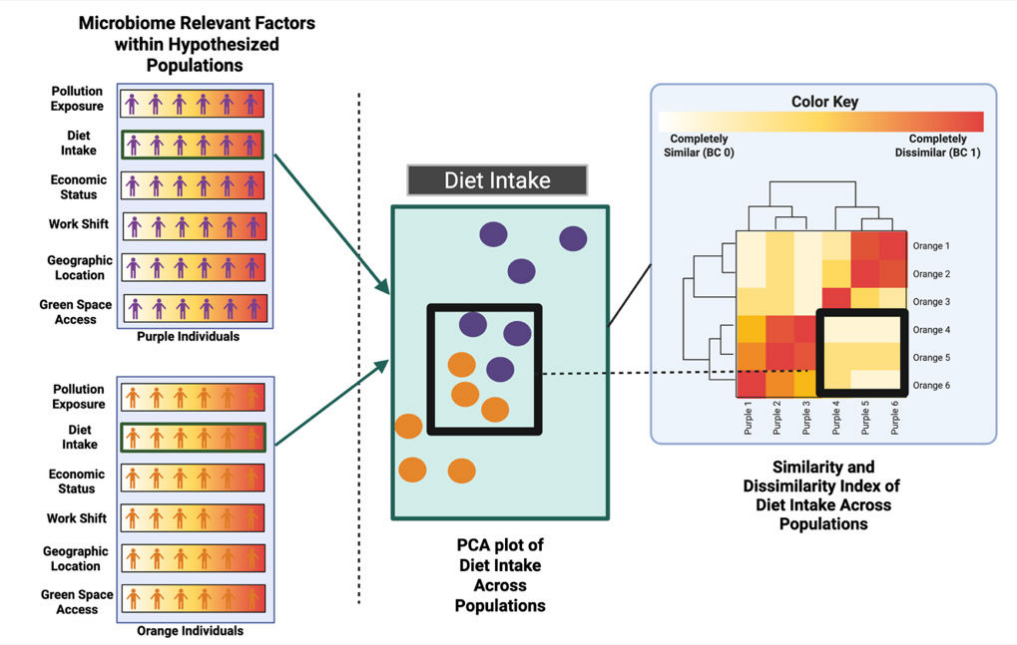
Far left panel: selected social and environmental factors that modulate the microbiome shown as continuums among two hypothesized populations (purple and orange people). Middle panel: selection of data from one factor (economic) showing economic variation within each population on the PCA plot. Far-right panel: similarity-dissimilarity index matrix of economic factors between the hypothesized groups. Research implications: if the study consists of these populations, then analysis primarily based on group categories of purple or orange would miss the potential contribution of economic factors between the groups. Under these circumstances, study population stratification by economic factors may provide better insight into microbiome modulation. Image created with BioRender.com.

The role of the environment is unrefuted in microbiome science, even in the presence of considering human genetic-microbiome connections (11, 80). Environmental factors relevant to the microbiome can include building materials, biodiversity, pollutants, social networks, and healthcare access. Differences in exposures to these microbiome-relevant factors are often present as a result of race-based policies, laws, and social norms that change risk of exposures based on social position, which, through these laws, policies, and norms, can become inextricably linked to racial groups. Thus, when considering race and ethnicity in human microbiome studies, providing contexts for environmental conditions that influence microbiome and disproportionately occur within racial and ethnic groups may provide an ecological-based frame for understanding mechanisms that may explain racial and ethnic-based differences in the microbiome. Recent literature has provided evidence for the role of these factors in microbiome composition and diversity (81–87). In addition, when a cohort lacks variation in social and environmental factors, the role of an ethnicity category in predicting microbiome is actually minimized (11). Figure 2 presents an array of ecological-based factors that may be considered social and environmental modulators of the microbiome that may be measured precisely and that could explain race or ethnic differences in microbiome composition or diversity. Validated measurement tools for many of these variables (Fig. 3) may be identified through cited references or from validated measurement databases, such as the PhenX toolkit (88). Finally, the selection of social and environmental factors is important and should be done with respect to research questions and with a discussion of the limitations of social and environmental measures. For example, a recent publication by members of this author group used a social vulnerability index to measure geographic differences in alpha-diversity (84). However, this index does not include measurement of food environments, thus potentially limiting insight into the gut microbiome diversity within the study.

Although disproportionate within racially marginalized communities, the presence of inequitable social and environmental factors is not exclusive to or monolithic within these communities. Therefore, identification of an individual or a study sample that includes persons who identify, for example, as Black, Hispanic, or Asian is not sine qua non with inequitable or disadvantaged environments. Likewise, inclusion of individuals who identify as White is not equivalent to high economic status in all cases. Social and environmental factors must be measured even in the presence of capturing race and ethnicity-based category data. Failure to include measurement of these factors when including race and ethnicity in studies may belie racialized stereotypes of low SES, poor education, or even poorer health among racially marginalized individuals. Using a model of a PCA plot from hypothesized populations (orange and purple individuals), we offer an example for understanding the role of continuums that can exist within a population group (Fig. 3) for modulators of the microbiome. Due to this variation potential, when conducting between-group analyses, we encourage researchers to identify when factors are similar or dissimilar between groups and not presume that group categorization signifies uniformity of environmental factor exposure (Fig. 3). Comparably, studies across cohorts should provide known and relevant factors such as SES for each cohort as differences in SES may confound microbial results. Furthermore, providing only race and sex/gender variables will not provide additional mechanistic explanations; for example, to date, the Human Microbiome Project and American Gut Project represent people with income levels that skew higher than average because of where participants were initially recruited from. Intersectionality, the interconnected nature of social categorizations, is non-additive (experiences are not linearly predicted) and non-separable (experiences based on gender and race are conjoined) (14, 89). This is especially germane to studies on the vaginal microbiome, which, when not placed in the context of measured social and environmental factors, may inadvertently reinforce narratives on race and female sexuality (14).

##### How to identify social and environmental factors

For researchers working on biological or genetic contexts, refer to NASEM population descriptors; otherwise, you are probably referring to social and environmental factors instead. The socioeconomic, cultural, environmental, and genomic considerations that shape human microbiomes are highly context-specific and require an in-depth understanding of the people you are working with. Most microbiome researchers are not trained in anthropology or bioethnography, and it can be difficult to acquire these skills within the timeframe set by many microbiome project periods, especially when working with demographics who are new to you, as it takes trust, ongoing conversations, and consistent interactions over time to build relationships with any demographics for the purpose of microbiome research. Although building these skills and these collaborations within the community is important for microbiome researchers to improve their own research and their ability to collaborate across disciplines, we find that it is critical for microbiome scientists to consult other experts. In particular, the context of race and racism is highly specific to geographic areas and cultural backgrounds, and this context may be too nuanced for researchers who are not specialized in social science research. Thus, to improve human microbiome research, which includes aspects of race, racism, or cultural effects on microbial communities, we strongly encourage microbiome researchers to collaborate across disciplines.

### Advance microbiome science through cooperative production of knowledge

#### We should learn to acknowledge the social impacts of publishing our work, not ignore them

*NASEM Recommendation 3: researchers, as well as those who draw on their findings, should be attentive to the connotations and impacts of the terminology they use to label groups.*

There are consequences to racializing bodies in science, and we can see this in historic examples—the misguided concept of phrenology, the demand for organs for transplant from people based on skin color, or the abuse of research participants of certain backgrounds, as well as in contemporary examples, including the NASEM report (90, 91).

Currently, racialization of bodies still occurs, and now, we have added the microbiome as a metric by which certain microbiomes may be labeled “undesirable,” as discussed in (92). For example, vegetables and dairy products are promoted as part of an idealized healthy diet in much of Western culture as a way to improve nutrient intake and recruit beneficial gut microbiota. However, in many geographic regions with limited food production or availability, dairy was not a part of the traditional diet, and most of the human population lacks the ability to produce the lactase enzyme to digest dairy products high in lactose. Instead, many cultures developed creative and skillful ways of improving nutrient content, storability, and harnessing beneficial microbiota (93). Idealized diets create harm when promoted through marketing and policy to the point of displacing local food systems, often leading to food intolerances and metabolic disease (94). Furthermore, the industrialization of local food systems has reduced the microbial diversity of foods (95).

Another widely discussed example (3, 4, 6) is early research on vaginal microbiomes, which connected one vaginal bacterial community state type to disease and to race (i.e., physical characteristics) (10, 96). This has been propagated in several studies, all of which use fewer than 50 individuals in a group (57), and which ignore geographic disparities in the subjects in favor of pseudo-racial categories created by cherry-picking random groups to compare, as well as the conflation of racial and ethnic categories used in the United States (56). Notably, the early work has since been negated by a large-scale study by those researchers who discuss the effect of lifestyle on the vaginal microbiome rather than racial categories (13), which other large-scale vaginal microbiome studies had already noted (97) and have since expanded on in a global data set (98) However, the initial work, in large part because vaginal microbiome studies were rare at the time and had an outsized impact, generated harm in practice as medical, hormonal, diet, or hygiene interventions are being recommended to make the vaginal microbiome into our idea of what a healthy community should be, even after it has been widely shown that vaginal community state type does not on its own indicate health or disease, nor are certain states limited to people of one skin color or other (36).

As microbiome researchers, it is our intent to report our findings and not to pass personal judgment on our study subjects, and we avoid adding social context because we lack the training to do so. However, when scientific study subjects or populations are categorized by socially defined race, the pervasive message (to the scientific readership and eventually the general public) is that race is so biologically essential that it even affects our microbes. If no interpretive guidelines are found in the published research, problematic assumptions or interpretations can spread unhindered.

Furthermore, microbiome researchers of today have inherited the consequences of institutional harm across generations, as many universities in the US were built with enslaved labor on stolen Indigenous land; discriminated against applicants, students, and faculty/staff based on race; used “othering” language to separate the on-campus population from the rest of the community; and encouraged researchers to avoid pointing to racism as the cause of educational or health disparities, as reviewed previously (37). Furthermore, redlining and other policies that segregate communities by skin color, including policies which were encouraged by universities (38), have encouraged educational or health disparities in many cities, such that researchers who focus on local populations without discussing the historical context of racism in their city risk obscuring the real cause of disparities in microbial exposure and microbiome function.

#### Reduce harm by adding awareness through context

Microbiome researchers spend quite a bit of time thinking about the correct terminology for the microbial communities we describe, such as their alpha and beta diversity, their change over time, and whether to call it a microbiome or if it is really just taxonomic information about a bacterial community. We should extend this same consideration to the framing of our biological and social variables as well, and it is important to do this in highly visible portions of articles in addition to the Methods or Discussion sections, which may be hidden behind “pay-walls.” For example, instead of using “race” in titles or abstracts, which are sometimes the only portions of publications or presentations that are read (9, 39, 56), use more precise terminology as discussed in the NASEM report. When considering ways to design research, a helpful exercise for authors is to imagine reading their paper or presenting the work to a room full of the study participants or sharing it on social media to an audience of laypersons relevant to the study. How might the author feel after such an exercise? How might the audience feel?

Studies on human microbiome and health, which point to the impacts of poverty, are useful in generating empirical data that support policy change; however, researchers preclude discussion by summarizing only that more research or solutions to ameliorate poverty are needed. The community that is being studied certainly already knew that and very likely could have pointed to possible solutions or barriers. One case study is Max Liboiron’s work on marine microplastics (40), in which the research team identified large quantities of microplastics in fish from an area used for subsistence fishing, which the local community was already aware of but lacked the resources to clean up the pollution coming in from other industries or communities. Before publishing the results, the research team met with the local community to discuss their findings and the impact that this information would have, for example, by instigating a policy to ban fishing from that area, resulting in a loss of that food source. Similar commitments to reciprocity and accountability have been demonstrated in other contexts, where investigators have partnered with Indigenous and urban communities to co-develop research priorities, establish frameworks for data governance, and ensure findings are translated in ways that serve community well-being. These approaches, which range from consensus processes that define health determinants to relational frameworks that embed Indigenous oversight of microbiome projects to perspectives that weave cultural health concepts with scientific inquiry, show how collaborative models can both advance science and protect community interests (41, 99, 100).

In publications, including methodologies for dissemination and impact summaries would enhance the visibility of these actions and improve research holistically. For example, including a paragraph on the impact of the study and the results on participants is something that is included in Institutional Review Board proposals, but not often propagated to the publication record, and there is usually no information about how study participants or the broader community were reached. Authors and researchers can further support improvement across the microbiome community through peer-review mechanisms. Peer reviews on manuscripts, contributed presentation abstracts, and funding proposals can all suggest reframing or ask for additional context as needed. Journal editors who handle human microbiome manuscripts may cultivate an interdisciplinary perspective by reaching out to anthropologists or social scientists to act as complementary peer reviewers to the microbiome reviewers who are already being consulted on the microbiological methodologies and interpretation of results.

Educators and mentors can have a direct impact on students and trainees who are forming their roadmap for how to conduct ethical and rigorous research. Incorporating social and environmental contexts into coursework on microbiology and/or health (46, 101–103) is critical to reminding students that we cannot understand microbiomes without understanding their host and the world around them.

As researchers, mentors, educators, students, and peer reviewers, we are all members of a racialized society and therefore susceptible to bias and social conditioning based on our own lived experiences with race, class, migration, and social position. Reflexivity may provide a method for scientists to become aware of their subjectivity and prior experiences. Although traditionally conducted within qualitative research, recent calls have occurred to consider the use of reflexivity within quantitative and the “hard” sciences (104). As scientists, we know that complex interactions and communications occur within the scientific process (i.e., conceptualizing study designs, selection of variables, and examination of analyzed data). Exploring how these interactions and communications represent our lived experiences and may impact the research process is important to strengthen the rigor of our field into the future. It is important to note that improving these processes will take intentional human effort. There is currently widespread conversation about how artificial intelligence (AI) and its associated technologies will play a role in scientific investigation. However, we want to caution against the presumption that replacing human decision-making with AI will fix the issues outlined above. It is crucial to recognize that AI, although powerful, cannot ameliorate centuries of deeply ingrained societal structuring and bias, in particular because these technologies are trained on existing data, including categorical metadata, unaddressed batch effects from meta-analyses, or a conflation of exposure and lifestyle choices for categorical demographics. Furthermore, we caution against delegating interpretive powers to AI: the digital landscape is flush with historical and contemporary information, including articles, studies, interpretations of studies, and data sets that perpetuate and even reinforce these problematic understandings of human difference (105). Without intervention, AI can and often does reproduce and amplify these existing biases (106–109). The complexity of how socially constructed realities impact our health requires a nuanced understanding that goes beyond purely computational pattern recognition (105). Scientific data forms the basis of clinical decision making, biomedical innovation, and investment with global impact. We must not delegate our responsibility elsewhere.

#### Seek expertise through collaboration, rather than “reinventing the wheel”

*NASEM recommendation 5: researchers, especially those who collect new data or propose new courses of study for a data set, should work in ongoing partnerships with study participants and community experts to integrate the perspectives of the relevant communities and to inform the selection and use of population descriptors.*

Similarly, the phrase “nothing about us without us” has been a rallying cry for researchers to include study populations as partners in the generation of knowledge and development of policy. This is practiced in medicine (110, 111), and there are now numerous resources on developing ethical human microbiome research and data sovereignty (6, 112–114), as well as justice-based microbiome study (115).

However, microbiome researchers are not often offered training in community-based research or anthropology/sociology while simultaneously being pushed by institutions and funders to have multiple expertise within their lab. Sometimes microbiome research is not seen as an independent field of study and is viewed as just a tool to be applied in combination with “real” research methodologies. Rather, researchers must accept that microbiome science, which embodies microbiota, host, and environmental factors, is inherently interdisciplinary and be reminded that it is ok to ask for help. Despite being practiced for decades, it is still a novel idea for microbiome researchers to work with anthropologists instead of feeling pressured to collect categorical population descriptor data and behavioral survey data on our own. Or microbiome researchers are taught that anthropologists are not needed unless they are studying non-industrialized populations far away.

There are demonstrable benefits to and guides for establishing interdisciplinary collaboration (46). There may be appropriate use of race and ethnic characterizations when microbiome research is exploring the role of human microbiome composition, impacts of racism/discrimination, and racial/ethnic health disparities, similar to the NASEM Report’s Table S1 (page 14) (2). Due to its connection with human physiology and function and to social and environmental factors, the microbiome may become a tool for not just documenting health disparities but resolving health inequities. In order to make this important contribution to public health, it is imperative that microbiome studies include socially, biologically, and economically diverse participants within studies as a best practice within the field. The inclusion of diverse participants may additionally better aid the field in expanding identified social and environmental modulators of the human microbiome.

#### Expand your research network to include social, economic, or political context expertise

If you are connecting to other researchers, there are challenges that come up simply because of discipline-specific training. For example, jargon and field-specific terminology for the same concept can be a barrier to communication, and different styles of professional writing can create obstacles to forming tangible outputs. However, overlooked aspects include different turnaround times for projects, what constitutes a meaningful end goal, and expectations in different disciplines. Be sure to discuss in advance any institutional requirements (e.g., publication style or venue) that may need to be reviewed by both institutional heads; some offices may have stringent conditions. Luckily, the solutions to most of these problems are good communication and relationship skills. You need to talk to each other often, set roles and responsibilities early, and be more specific than you might be with a colleague who does the same work as you. Being flexible about timelines and end goals is important to make sure everyone benefits in a timely manner, and making sure to connect resources can also help the group stay coordinated.

A case study example of this is the Microbes and Social Equity (MSE) working group, which was formed in 2020 with the purpose of connecting microbiology with social equity research, education, policy, and practice to understand the interplay of microorganisms, individuals, societies, and ecosystems. MSE’s goals are to generate and communicate knowledge that will spark evidence-based public policy and practice, supporting equity and sustainability for all. MSE includes members who “represent diverse fields, for example, anthropology, architecture, bioethics, bioinformatics, data science, ecology, engineering, genetics, medicine, microbiology, nutrition, psychology, and sociology, and exhibit expertise in various hosts, systems, and environments beyond human microbiomes. We are researchers, educators, practitioners, and policymakers spanning the globe and career levels.” (116). To that end, MSE has hosted annual seminar series and symposia, as well as a journal special collection, featuring topics on biopolitics (116); income inequality and the microbiome (117); racism and the microbiome (100, 118), especially through policies restricting food systems (92, 119); creating context-aware experimental designs (118); and creating community engagement to mutually generate benefits between researchers and the people whom they study (118). These peer-to-peer learning and discussion activities can help provide this context, training, and networking opportunities to improve human microbiome research (120).

## SUMMARY

The NASEM report is very comprehensive and gives best practices recommendations for the use of population descriptors in genetics and genomics research. Here, we took key principles and recommendations from the report and adapted them to the study of human microbiomes. This document should not be seen as exhaustive; rather, it should be seen as a clear initiation of a discipline-wide discussion from which we expect to refine our methods and produce rigorous science that moves our understanding forward.
